# DNA Dye Sytox Green in Detection of Bacteriolytic Activity: High Speed, Precision and Sensitivity Demonstrated With Endolysins

**DOI:** 10.3389/fmicb.2021.752282

**Published:** 2021-10-25

**Authors:** Marek Harhala, Katarzyna Gembara, Paulina Miernikiewicz, Barbara Owczarek, Zuzanna Kaźmierczak, Joanna Majewska, Daniel C. Nelson, Krystyna Dąbrowska

**Affiliations:** ^1^Laboratory of Phage Molecular Biology, Department of Phage Therapy, Hirszfeld Institute of Immunology and Experimental Therapy, Wrocław, Poland; ^2^Research and Development Centre, Regional Specialist Hospital in Wrocław, Wrocław, Poland; ^3^Bacteriophage Laboratory, Department of Phage Therapy, Hirszfeld Institute of Immunology and Experimental Therapy, Wrocław, Poland; ^4^Institute for Bioscience and Biotechnology Research, University of Maryland, Rockville, MD, United States

**Keywords:** endolysin, kinetic, Sytox Green TM, bacterial detection, bacterial lysis, bacteriolytic activity, lysins, fluorescent DNA dye

## Abstract

**Introduction:** Increasing number of deaths from multi-drug resistant bacterial infections has caused both the World Health Organization and the Centers for Disease Control and Prevention to repeatedly call for development of new, non-traditional antibacterial treatments. Antimicrobial enzymes, including those derived from bacteriophages, known as endolysins or enzybiotics, are considered promising solutions among the emerging therapies. These naturally occurring proteins specifically destroy bacterial cell walls (peptidoglycan) and as such, are capable of killing several logs of bacteria within minutes. Some endolysins cause lysis of a wide range of susceptible bacteria, including both Gram-positive and Gram-negative organisms, whereas other endolysins are species- or even strain-specific. To make wide use of endolysins as antibacterial agents, some basic research issues remain to be clarified or addressed. Currently available methods for testing endolysin kinetics are indirect, require large numbers of bacteria, long incubation times and are affected by technical problems or limited reproducibility. Also, available methods are focused more on enzymatic activity rather than killing efficiency which is more relevant from a medical perspective.

**Results:** We show a novel application of a DNA dye, SYTOX Green. It can be applied in comprehensive, real-time and rapid measurement of killing efficiency, lytic activity, and susceptibility of a bacterial population to lytic enzymes. Use of DNA dyes shows improved reaction times, higher sensitivity in low concentrations of bacteria, and independence of bacterial growth. Our data show high precision in lytic activity and enzyme efficiency measurements. This solution opens the way to the development of new, high throughput, precise measurements and tests in variety of conditions, thus unlocking new possibilities in development of novel antimicrobials and analysis of bacterial samples.

## Introduction

Bacterial infections were the main causes of death worldwide before the 20th century, and remain leading causes of death in low-income countries (Department of Commerce and Labor B of the C, 1906; Armstrong et al., 1999; World Health Organisation (WHO), 2018b). The threat of antibiotic resistance began with the first use of antibiotics themselves (Fleming, 1945; Nikaido, 2009). The increase in the number of deaths due to bacterial infections in the US and other high-income countries since the 1980s, combined with the emergence of multi-drug resistant strains spreading throughout the world, suggests that the age of efficient antibiotics has come to an end (Department of Commerce and Labor B of the C, 1906; Armstrong et al., 1999; Hoyert et al., 1999; Nikaido, 2009; Hernando-Amado et al., 2019). As a result, the World Health Organisation (WHO) and the Centers for Disease Control and Prevention have repeatedly called for development of new or alternative antibacterial treatments (World Health Organisation (WHO), 2001; United States Government, 2015; World Health Organisation (WHO), 2015; 2016; 2018a).

One antimicrobial approach that has gained increasing recognition is the use of various enzymes, called enzybiotics, that show the potential to limit threats posed by bacteria (Labrou, 2019; Liu et al., 2019; Song et al., 2020). Enzybiotics are fast-acting proteins that hydrolyze the peptidoglycan, a distinctive structural element of bacterial cells, resulting in osmotic lysis and cell death (Young, 1992; Fischetti, 2001). The vast range of possible enzybiotics provides a high chance of finding or engineering these enzymes to meet therapeutic needs. Among enzybiotics, endolysins are a specific group that are derived from bacteriophage (phage). Endolysins have been shown capable of lysing almost all bacteria, including multi-drug resistant bacteria, often in a span of a few minutes, and have been validated in animal models without detected toxicity (Loeffler et al., 2003; Harhala et al., 2018; Abdelkader et al., 2019; Dams and Briers, 2019). In 2019, phage endolysins were recognised by WHO as innovative, non-traditional biologicals in development (World Health Organisation (WHO), 2019). Notably, three endolysins are currently in Phase 2 or Phase 3 human clinical trials (Schmelcher and Loessner, 2021).

Discovery, characterization, and benchmarking of endolysins has been hampered by key technical issues. Typically, bacteriolytic activity testing utilizes a spectrophotometric turbidity reduction assay (TRA), which is an indirect simplification of complex physical phenomenon, including light scattering and refraction, when determining absorbance of a solution (Mitchell et al., 2010). This results in high variability between repeated measurements and limits the range of reaction environments that can be tested. Alternative methods include traditional testing for growth of surviving bacteria, but these assays can also be highly variable due to serial dilutions, do not provide real-time measurements, and are not amenable for medium- or high-throughput applications. Recently, lysis efficiency has been measured by detection of ATP released from bacteria (Heller and Spence, 2019), which requires shorter incubations, but it still does not provide results in real-time and can be confounded by bacteria that display glycolytic enzymes on their surface that can produce ATP (Fischetti, 2016).

In this study, we investigate DNA dyes as the source of a quantitative, real-time measurable signal of peptidoglycan destruction and bacteriolysis. These dyes do not require active bacterial growth or metabolism to induce the signal, which is detected immediately when bacterial DNA becomes available for binding by the dye in lysed bacterial cells. This simplifies the procedure and enables detection in difficult or complex samples, such as environmental or clinical isolates. Here, we show the applicability of a fluorescent dye for a real-time detection of bacterial cell disruption and we use this new method to characterize Cpl-1 and Pal, two endolysins active against *Streptococcus pneumoniae*.

## Materials and Methods

### Protein Expression

The endolysins, Pal (Acc. no. YP_004306947), derived from *Streptococcus* phage Dp-1, and Cpl-1 (Acc. no. CAA87744), derived from *Streptococcus* phage Cp-1, were used in this study. The Cpl-1 and Pal coding sequences were cloned into the Gateway cloning system, then amplified with primers incorporating a C-terminal 6xHis tag and subcloned into a pBAD24 expression plasmid. These vectors were expressed in *E. coli* B834(DE3) cells (EMD), at 37°C with shaking in Luria-Bertani (LB) broth (10 g/L tryptone, 10 g/L NaCl, 5 g/L yeast extract) supplemented with ampicillin (50 mg/L) (Sigma-Aldrich, Europe), until the OD_600_ reached 1.0. Next, the bacterial culture was cooled to 22°C and protein expression was induced by the addition of arabinose at a final concentration of 2.5 g/L. The culture was incubated overnight at 22°C with intensive shaking.

### Protein Purification

Bacteria were harvested by centrifugation (8,000×*g*, 10 min, 4°C), resuspended in PBS (50 mM Na_2_HPO_4_, 300 mM NaCl, pH 7.2) in 1/10th volume of the culture and PMSF (1 mM) and lysozyme (0.5 mg/mL) were added. The mixture was incubated for 2 h on ice with gentle shaking and lysis was achieved using the freeze-thaw method. Mg^2+^ (up to 0.25 mM), DNAse (20 mg/L), and RNAse (40 mg/L) were then added to the extract and allowed to incubate on ice with gentle shaking for 3 h. The fractions were separated by centrifugation (12,000 × *g*, 30 min, 4°C) and the soluble fraction (supernatant) was collected. PBS buffer containing 500 mM imidazole was added to the soluble fraction to adjust the final concentration of imidazole to 50 mM. The target proteins were bound to NiNTA agarose (Qiagen, Hilden, Germany) at room temperature and washed with PBS (6 × volume of the agarose) with increasing concentrations of imidazole (75, 100, 250, and 500 mM). The 100 and 250 mM imidazole fractions contained the eluted endolysins and these fractions were dialyzed against PBS at 4°C with a 3 kDa cut-off membrane (SpectraPor). Next, LPS removal was performed with an EndoTrap Blue column (Hyglos GmbH, Munich, Germany). To complete purification, samples were dialyzed dialyzed (3,000 kDa molecular weight cut-off, 3 times, 2,000 mL bottles for 20 mL of protein sample, 4°C, Spectra/Por, Repligen) against PBS and filtered through sterile 0.22-μm polyvinylidene difluoride filters (Millipore, Burlington, MA, United States). The process was monitored by SDS-PAGE at all stages. Protein concentration was determined by the Bradford assay (Sigma-Aldrich, Europe) following the manufacturer’s instructions.

### Testing Activity, Fluorometric Assay

Sytox Green solution from ViaGram^TM^ Red+ Bacterial Gram Stain and Viability Kit (Thermo Fisher Scientific) was used in this article. Kinetic experiments were performed at room temperature (23°C). Several controls were used to ensure technical suitability and reproducibility of experiments. Briefly (detailed description below), in each experiments we measured fluorescence of PBS (technical control), killed bacteria with isopropanol (positive control), live bacteria only (negative control), lytic agent only (enzyme control) and bacteria killed by endolysins (lysis control) showing the level of fluorescence when lytic reaction is complete.

The *technical control* (background) measures fluorescence of the background coming from the buffer, the plate and any unbound fluorescent dye. It consists of 150 μL PBS mixed with 50 μL of diluted (1/100) Sytox Green solution. The mean signal of this control was subtracted from all reads.

The *positive control* is used to demonstrate that the DNA dye in the sample is of sufficient quantity (e.g., not saturated) and the measurements are valid. This control is comprised of a concentrated suspension of dead bacteria that gives extremely high fluorescent reads. The signal measured by this control sample should be higher than in any other sample, establishing that the DNA dye is not saturated in the experimental groups and are technically valid. Preparation of the concentrated suspension of dead bacteria is based on a protocol provided by manufacturer of the DNA dyes (Thermo Fisher). Briefly, 1 mL of *S. pneumonia* grown for 22 h at 37°C in Todd-Hewitt broth without shaking was added to 20 mL of 70% isopropyl alcohol and incubated for 1 h at room temperature with mixing every 15 min, followed by centrifugation (15,000 × *g*, 15 min, 4°C) and resuspension in 0.25 mL of PBS. 50 μL of this suspension was mixed with 50 μL of diluted (1/100) Sytox Green and 100 μL of PBS.

The *negative control* (bacteria) included 100 μL PBS with 50 μL of diluted (1/100) Sytox Green and 50 μL of previously prepared bacteria (OD_600_ = 1.0). This control was used for normalization to calculate progress equal to 0.0 for each timepoint.

The *enzyme control* consists of 100 μL PBS with 50 μL of diluted (1/100) Sytox Green and 50 μL of previously prepared enzyme solution in PBS (at the highest concentration used for the experiment).

The *lysis control* (for calculation of *max* signal in normalization) contains 50 μL PBS, 50 μL of diluted (1/100) Sytox Green, 50 μL of previously prepared enzyme (80 mg/L for Cpl-1 and 40 mg/L for Pal enzyme) and 50 μL of bacteria solution. The *max value* is defined as the mean signal measured in the last 3 min of the reaction. If lysis has gone to completion, the progress curves should show no statistically significant change in the last 3 min (i.e., the curve is flat). Enzyme concentrations included in the experiments higher than proposed can be used for calculation of the *max signal*. The amount of lysin used for this control was chosen such that complete lysis (99.9%) occurred within 10 min. Since activity of the bacteriolytic agent depends on bacteria strain, amount of the bacteriolytic agents needs to be sufficient for complete lysis in 10 min. For experiments presented in this article, the lysis of more than 99.9% of bacteria was confirmed by dilution testing.

#### Before Experiment

##### Each sample is tested in triplicate

Prepare concentrations of the enzymes remembering that for a typical assay the enzyme is twice diluted after being added to the sample, so stock solutions of the enzyme needs to be twice the final desired concentration.

#### Bacteria Preparation

Susceptible bacteria of *S. pneumoniae* were grown for 20 h at 36°C in (50 mL) in Todd-Hewitt broth, no shaking.

Bacteria were harvested by centrifugation (7 000 × g, 7 min, 20°C).

Supernatant was removed.

Pellet was resuspended gently in PBS (25 mL).

Sample was centrifuged again (7,000 × g, 7 min, 20°C)

Pellet was resuspended gently in PBS (25 mL).

Sample was centrifuged again (7,000 × g, 7 min, 20°C)

Pellet was resuspended in PBS to final OD_600_ 1.0.

The sample was incubated for 30 min at a room temperature.

The bacterial sample is now ready for fluorescent measurements.

#### Controls Preparation (Each Should Be in Triplicate per Plate)

Positive control (should be started 2 h before the experiment):

Sytox Green solution from ViaGram^TM^ Red + Bacterial Gram Stain and Viability Kit (Thermo Fisher, alternatively – 5 mM solution of Sytox Green in DMSO can be used) was diluted in PBS 100 times (volume needed is 50 μL per well).

10 mL of bacterial sample from (see Bacteria preparation section) were centrifuged (7 000 × g, 4°C, 7 min).

The supernatant was discarded.

The pellet was resuspended in 1 mL of PBS.

20 mL of 70% isopropyl alcohol was added (water solution).

The sample was incubated 1 h at room temperature and mixed every 15 min.

The sample was centrifuged (15 000 × g, 15 min, 4°C).

The supernatant was discarded.

The pellet was resuspended in 350 μL of PBS.

50 μL of PBS with diluted Sytox Green and 100 μL of resuspended pellet were mixed in the well before the experiment.

Technical control: 150 μL of PBS and 50 μL of diluted Sytox Green solution was mixed.

Lysis control: 50 μL of PBS, 50 μL of diluted Sytox Green solution, 50 μL of bacterial sample and 50 μL of endolysin (at least 80 μg/mL for Cpl-1 and 40 μg/mL for Pal) was mixed.

Enzyme background control: 100 μL of PBS with 50 μL of diluted Sytox Green solution and 50 μL of endolysin (equal to highest concentration used in the experiment) was mixed.

Negative control (bacteria without endolysin): 100 μL of PBS, 50 μL of diluted Sytox Green solution and 50 μL of bacteria was mixed.

#### Fluorescent Measurement

To each well, 50 μL of diluted Sytox Green solution and 100 μL of endolysin stock solution in PBS (two times higher than tested) was added.

All necessary controls were prepared and mixed.

Enzymatic lysis was started by adding 50 μL of bacteria suspention to each well.

Immediately start reading the signal in each well (ex./em.: 475/525 nm) every 45–60 s.

Measurements continued for at least 10 min.

Validity of the experiment – results were considered valid only if:

Average raw signal of technical control is lower than the average signal enzyme control.

Average raw signal of the enzyme control is lower than average signal of negative control.

Average raw signal of positive control yields at least 33% higher reads than any other sample in the experimental setup.

Average raw signal from lysis control from last 3 min of the experiment shows no statistically significant change after the initial rise.

### Testing Activity, Turbidity Reduction Assay (TRA)

As a comparison for the fluorometric assay, turbidity reduction assays were performed in parallel. The same protocol was followed with only the diluent (DMSO, 1% dilution in PBS) used in place of the solution of fluorescent dye. Otherwise, the same freshly prepared bacterial cells were utilized as was the same concentration range of endolysins. *Technical* and *negative* controls were the same as in the fluorometric assays. Samples were tested in standard 96-well plates in triplicate and absorbance readings were measured every for 10 min at 600 nm.

### Normalization and Computational Methods

Raw data from fluorometric assay and TRA can be normalized to an objective value: *progress* [of the lysis]. This is required for comparison between experiments using different measurement methods, enzymes, concentration of bacteria and/or enzymes. The *progress* (*x*) is defined as a part of the bacterial sample that is lysed, with values ranging between 0 (no lysis) and 1 (complete lysis of 100% bacterial cells). *Progress* is calculated by equation (1). Lysis control was used to calculate *max* signal for the purpose of normalization.


(1) $$x\,\left(t\right)=\frac{\left|r\,\left(t\right)-b\,c\,\left(t\right)\right|}{\left|m\,a\,x-b\,c\right|}$$

*x*(*t*) – progress at time t *r*(*t*) – signal of the sample at time t*bc*(*t*) – signal of bacterial control at time t*bc* –signal of negative control at 10 min (end of the experiment)*max* – signal of lysis control (enzymatically lysed bacteria)

*Progress* values can be used to calculate *lytic activity* as part of the total bacterial sample that is lysed per unit of time [min^–1^]. The Cpl-1 lysin demonstrated a *lytic activity* that fulfils the requirements of a linear regression model. This model was calculated from progress values 0.2 to 0.75 (for more detailed description see Supplementary Figures 1, 2A). In contrast, a one-phase association model best fit the data for the Pal endolysin (GraphPad Prism 7; *R*^2^ > 0.99 for both measurement methods) from 1 to 110 mg/L. *Lytic activity* in this model is evaluated by a *K* value, which depicts the change of *progress* at the beginning of the reaction (i.e., the initial velocity) (see Supplementary Table 1 and Supplementary Figure 2B). In low concentrations of endolysins (below 1 mg/L of Pal and below 2 mg/L for Cpl-1), all measured points were used for calculation of linear regression due to insufficient lysis by the end of the 10 min assay. In these cases, the measured slope was used to define the lytic activity. Comparison of the mathematical models are summarised in Supplementary Table 2. A dilution test was used to calculate number of bacteria (as CFU) corresponding to OD_600_ values.

### Bacteria Used for Lytic Experiments

Clinically isolated *Streptococcus pneumoniae* strain (code PN135) was used for all experiments. It is susceptible to optochin, Pal and Cpl-1.

## Results

### Evaluation of Sytox Green as a Detector of Bacterial Lysis in Real Time

**FIGURE 1:**
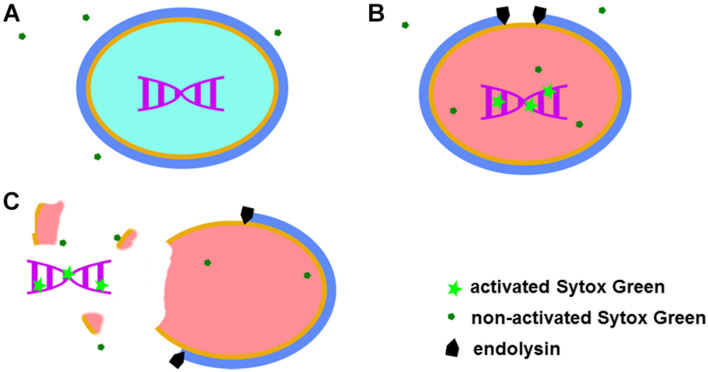
Graphical representation of the Sytox Green mode of action. When added to a bacterial mixture, Sytox Green is excluded from metabolically active (live) bacteria and DNA remain sequestered inside the bacterial cell resulting in no signal **(A)**. When a bacteriolytic agent (e.g., endolysin) is added to a mixture of live bacteria and Sytox Green, lysis of the bacterial cell begins. The bacterial cell becomes metabolically inactive and dies when sufficient lysis occurs, allowing Sytox Green to enter the cell ghost and bind to DNA resulting in increased fluorescence **(B)**. In the case of extensive damage or even complete disintegration of the bacteria, bacterial DNA can leak outside the cell and Sytox Green in the mixture binds the DNA producing a signal **(C)**.

Sytox Green fluorescent dye emits a signal after binding to double stranded DNA. This specific dye is also excluded from entering metabolically active bacteria (Figure 1A). Taken together, observed fluorescence after addition of Sytox Green dye can arise from three sources: metabolically inactive (dead) bacteria, DNA contamination in the sample and background fluorescence. After addition of a bacteriolytic agent such as an endolysin, live bacteria are lysed (killed) and their DNA becomes available to Sytox Green. An increase in fluorescent signal that follows such lysis therefore directly corresponds to the activity of the bacteriolytic agent (Figure 1).

Sytox Green proved to be the most suitable dye for measurement of bacterial lysis out of the four tested DNA dyes, including SYTO9, DAPI, propidium iodide, and Sytox Green (for details see Supplementary Figure 3 and Supplementary Table 3). We chose Sytox Green and compared our results to the turbidity reduction assay (TRA), the most commonly used assay for measuring endolysin activity. In short, after addition of an enzyme to a bacterial suspension, absorbance of light passing through the sample is measured at 600 nm and a drop in the absorbance is considered an indirect sign of bacterial lysis. Cpl-1 and Pal phage endolysins were chosen for these tests. Representative data are shown in Supplementary Figure 1 and results of data normalization are shown in Supplementary Figure 2. All data was normalized for comparison purposes based on controls as described in the “Materials and Methods.”

### Fluorometric Assay Measures *Lytic Activity* of Endolysins With Higher Responsiveness and in a Wider Range of Enzyme Concentrations Than the Turbidity Reduction Assay

**FIGURE 2:**
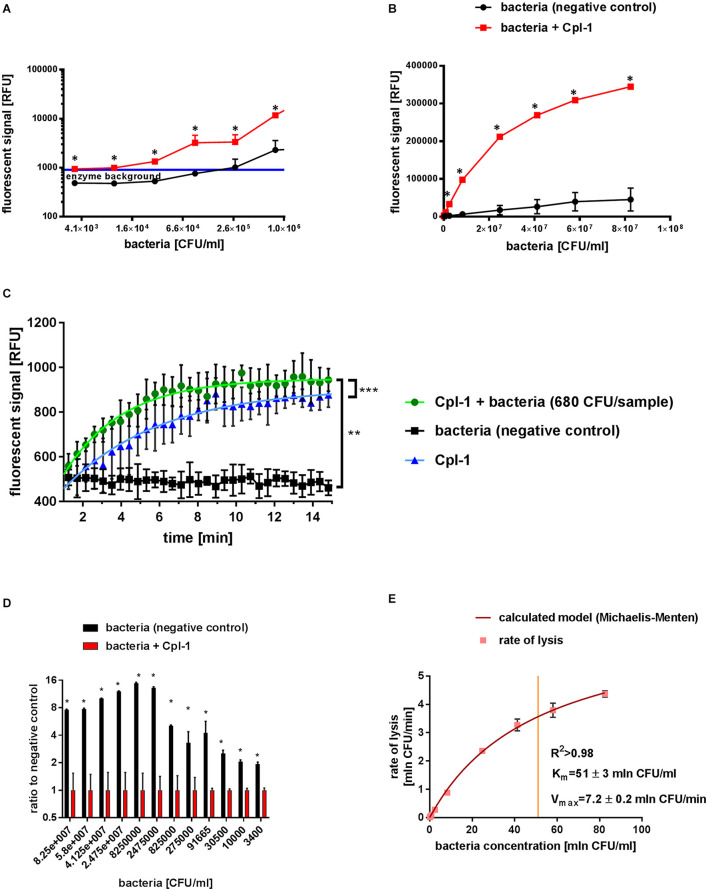
Characteristics of Cpl-1 activity by fluorometric Sytox Green assay. The fluorescent signal was measured after 10 min lysis by Cpl-1 (5 mg/L) over a range of bacterial concentration from 1.6×10^7^ CFU/sample to 6.8×10^2^ CFU/sample. Bacterial lysis was detected in PBS (200 μL) with bacteria at indicated concentrations and 0.25% Sytox Green solution. Fluorescent signal of Sytox Green after 10 min of bacterial lysis in low **(A)** and high **(B)** concentrations of bacteria (red) and background signal of bacteria with no endolysin (black). Blue line shows background signal of Cpl-1 alone. Kinetics of Cpl-1 activity in the lowest concentration of bacteria (680 CFU/sample) in comparison to background signals from bacteria (negative control) and Cpl-1 **(C)**. Ratio of signals from Cpl-1-lysed bacteria to negative control (bacteria) tested in a range of bacterial concentrations; the highest values represent optimal bacterial concentrations. **(D)** Graphical representation of a Michaelis-Menten model calculated for the Cpl-1 endolysin with experimental data **(E)**. * – statistically significant difference between samples and background (*p* < 0.003). ** and *** signifies statistically significant difference between *K* values of plotted curves (** is *p* < 0.0001, and *** is *p* = 0.0269). Points or bars represent average of measurements for two independent experiments and whiskers represent the standard deviation.

We present *lytic activities*, relative *lytic activities*, and specific *lytic activities* calculated from both fluorometric and turbidity reduction assays (TRA). Results are summarized in Figure 2 and the lytic activity is expressed as progress (part of the bacterial sample lysed per minute). The relative *lytic activity* is *lytic activity* presented as a percentage of *lytic activity* detected by the turbidity reduction assay under the same conditions. Specific *lytic activity* represents activity of 1 nmol of the endolysin in a specific experimental setup.

**FIGURE 3:**
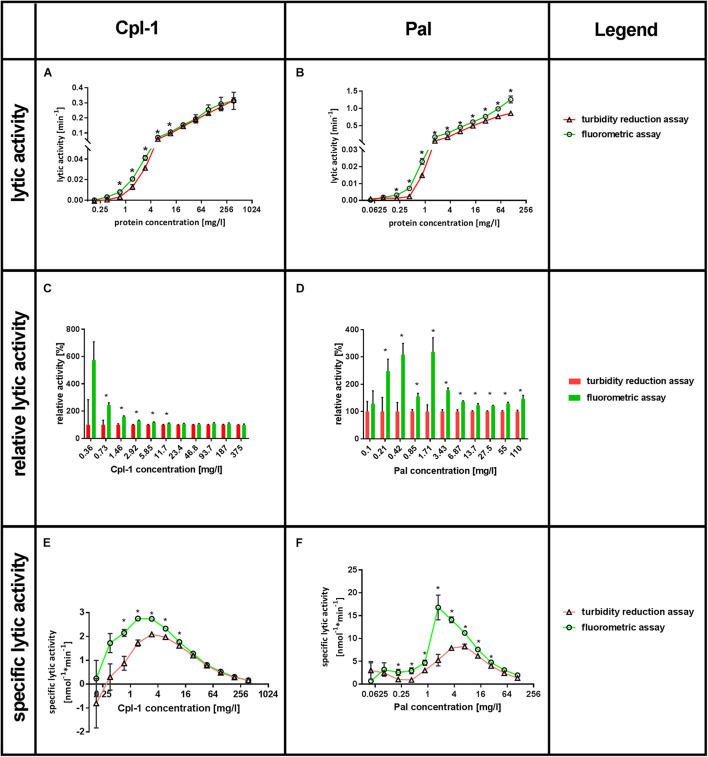
Comparison of Sytox^TM^ Green fluorometric assay and turbidity reduction assay in characterisation of Cpl-1 and Pal. Bacterial lysis was detected in PBS (200 μL) with 1.6×10^7^ CFU, Cpl-1 or Pal at indicated concentrations and 0.25% DMSO or Sytox Green solution (turbidity reduction or fluorometric assay, respectively, see Materials and Methods for details). Lytic activity of Cpl-1 **(A)** and Pal **(B)** is measured by both methods in concentrations ranging from 0.05 to 110 mg/L for Pal and from 0.18 to 375 mg/L for Cpl-1. Comparison of relative lytic activities for Cpl-1 **(C)** and Pal **(D)**. Specific lytic activity of Cpl-1 **(E)** and Pal **(F)** measured by both methods. (*) Stars show statistically significant difference in activity as detected by the two methods (adj. *p* < 0.05). Points represent results of two identical experiments. Bars represent the standard error.

*Lytic activity* was tested in a range of **enzyme concentrations**, ranging from 0.18 to 375 mg/L for Cpl-1 and 0.05 to 110 mg/L for Pal. *Lytic activity* was detected by Sytox Green as low as 0.36 mg/L for Cpl-1 and 0.1 mg/L of Pal, while the minimum concentrations shown to have activity with the turbidity reduction assay were four times higher. These values were found significant in comparison to negative control (bacteria, no bacteriolytic agent) (*p* < 0.001 and *p* < 0.008, for Cpl-1 and Pal, respectively). The *Lytic activity* detected by fluorimetric assay was significantly higher than that detected by turbidity reduction assay, thus demonstrating higher responsiveness of the Sytox Green-based method. For Cpl-1, statistically significant differences between assays were observed only at concentrations 11.7 mg/L and lower (*p* < 0.05) (Figure 3A), whereas significance between assays was detected in all concentrations of Pal (*p* < 0.05) (Figure 3B).

Relative lytic activity allows for comparison of the two methods by artificially setting the activity detected by the turbidity reduction assay to 100% for a given set of conditions (Figures 3C,D). In doing so, differences in lytic activity at lower concentrations of a bacteriolytic agent are revealed. Such differences might be important in situations where only low concentrations of an enzyme are possible (e.g., due to side effects, cost, poor penetration to the target site). This analysis also revealed that *lytic activity* detected by fluorometric assay was significantly higher than that detected by turbidity reduction assay, demonstrating higher responsiveness of the Sytox Green-based method. Statistically significant differences between assays were observed for Cpl-1 only at concentrations 11.7 mg/L and lower (*p* < 0.05) (Figure 3C), while significant differences were detected for Pal in all concentrations (*p* < 0.05) (Figure 3D). Analysis of correlation between relative *lytic activity* and enzyme concentrations for Cpl-1 revealed negative correlation (*p* < 0.0001, Spearman correlation). However, the correlation for the Pal endolysin was not observed (*p* > 0.18, Spearman correlation), therefore, we calculated average relative *lytic activity*, which was 180 ± 23% (*p* < 0.005).

The specific *lytic activity* as revealed by fluorometric assay and turbidity reduction assay differed markedly (Figures 3E,F). The fluorometric assay detected up to 2.44 times higher specific activity for Cpl-1 and 3.19 times higher for Pal in comparison to the turbidity reduction assay depending on the enzyme concentration. A drop in specific *lytic activity* at low enzyme concentrations are related to detection limits for concentrations below 0.36 mg/L for Cpl-1 and 0.1 mg/L of Pal. Conversely, a drop in specific *lytic activity* at high enzyme concentrations (Figures 3E,F) likely relates to inhibitory effects due to many endolysin molecules binding the bacterial surface despite only a single lytic event possible for each bacterial cell.

### Fluorometric Assay With Cpl-1 Detected Bacterial Lysis in a Wider Range of Bacterial Concentrations Than the Turbidity Reduction Assay, to as Low Concentration as 3.4×10^3^ CFU/mL

The *lytic activity* of Cpl-1 was detected in all measured bacterial concentrations, from as low as 3.4×10^3^ CFU/mL (6.8×10^2^ CFU/sample) to 8×10^7^ CFU/mL (1.6×10^7^ CFU/sample) in the fluorometric assay (Figures 2A,B). In contrast, optical density based methods, including the turbidity reduction assay, struggle to measure bacterial concentrations lower than 10^6^ CFU/mL (McKellar and Knight, 2000; Lewis et al., 2014; Yu et al., 2014; Meyers et al., 2018). Correlation between bacterial concentration and the detected signal of lysis was very high (0.9989, *p* < 0.0001 when 1.0 describes ideal correlation, Spearman correlation, GraphPad Prism 7) (Figures 2A,B).

We assessed potential dependence of Cpl-1 *lytic activity* detected by fluorometric assay on bacterial concentrations, and we found no statistically significant correlation (*p* > 0.2). Mean *lytic activity* was constant for all bacterial concentrations exceeding 5×10^3^ CFU/mL, not deviating from 0.192 (95% CI from 0.1849 to 0.1985 min^–1^; *p* < 0.01, GraphPad Prism 7, for details see Supplementary Table 4). However, *lytic activity* of Cpl-1 in bacterial concentrations lower than 5×10^3^ CFU/mL deviated from the calculated linear regression model, but a one-phase association model for this data was statistically significant (see Supplementary Table 5). This effect likely resulted from a relatively large contribution of signal generated by the endolysin in comparison to much lower contribution of signal from the low concentration of bacteria (Figure 2C). This contribution is a background signal from protein added at the start of the lytic reaction. We speculate it is a result of non-specific interactions between the protein and the dye. Nonetheless, this contribution is lower than 1/1000th of the activated dye and is considerable only in this extremely low concentration of bacteria. Thus, this model was used for evaluation of *lytic activity* at the lower limit of bacterial concentrations (Figure 2C).

### Optimal Concentration of Bacteria for Fluorometric Assay Was Between 2.5×10^7^ and 2.5×10^6^ CFU/mL per Sample

The optimal concentration of substrate bacteria was determined experimentally to further improve experimental design for practical applications. We calculated the signal to noise ratio for different bacterial concentrations for the Cpl-1 endolysin. We propose optimal range in presented experimental setup to be between 5×10^6^ and 5×10^5^ CFU, where the final signal reached over 12 times the background signal value (Figure 2D).

### The Cpl-1 Endolysin Is Characterized by Michaelis-Menten Model

*Lytic activity* of the Cpl-1 endolysin in varying concentrations of bacteria was used to calculate Michaelis-Menten statistics to develop a molecular characterization of the enzyme. We show that Cpl-1 can be described by the Michaelis-Menten model when measured by the fluorescent method. V_max_ represents theoretical upper limit for activity of the enzyme in an abundance of substrate. For Cpl-1, this was 7.2 ± 0.2 × 10^6^ CFU/min. The K_m_ represents the concentration in which an enzyme demonstrates half of its maximum activity. For the Cpl-1 endolysin, it was 51 ± 3×10^6^ CFU/mL (Figure 2E).

## Discussion

We evaluated the applicability of a DNA dye, Sytox Green, for detection of bacterial lysis. Our evaluation included two phage endolysins, Cpl-1 and Pal, both bacteriolytic toward *Streptococcus pneumonia*, varying concentrations of enzymes and bacteria. Taken together, we propose a new method for the detection of bacterial lysis and for characterization of bacteriolytic agents. This solution provides a very high sensitivity and is able to generate detectable signal even at concentrations of bacteria as low as 3.4×10^3^ CFU/mL (6.8×10^2^ CFU/sample). Importantly, the method offers direct, real-time measurements of reaction kinetics, thus allowing for calculation of *progress* and *lytic activity* parameters. We propose a mathematical approach to evaluate *lytic activity* measured from raw reads and we demonstrate its applicability in varying concentrations of two endolysins and bacteria. To demonstrate the potential of Sytox Green, we compared our results to results acquired in commonly used turbidity reduction assays, which detect absorbance of 600 nm light passing through a sample whereby a drop in the absorbance is considered an indirect indicator of bacterial lysis. As demonstrated in our study, use of DNA dye allows for detection of higher *lytic activity* of both enzymes, is more responsive and efficient (Figure 3), and capable for measuring *progress* of bacterial lysis in very low bacterial concentrations (Figure 2), thus having potential for detection of differences non-detectable for indirect turbidity assays like TRA.

Limitations for this fluorometric assay can be estimated from data presented in Figures 2, 3. In the tested conditions, activity can be detected in enzyme concentration as low as 0.36 mg/L for Cpl-1 and 0.1 mg/L for Pal. The maximum detectable activity was over 1.0 min^–1^ for the Pal enzyme, which can be interpreted as complete lysis in less than 2 min. The highest measured concentration of bacteria is over 8×10^7^ CFU/mL (0.2 mL volume of the sample) and lowest concentration of bacteria is 3.4×10^3^ CFU/mL (680 CFU in 0.2 mL volume of the sample). We speculate that lower concentration of bacteria can probably be measured by extending the monitoring time. All mentioned limits are, however, marked improvements in comparison to TRA. Additionally, a fit of a mathematical model to describe how the Pal enzyme (one-phase association) changes in response to different concentrations of bacteria has not presently been tested, but represents the next logical extension of this assay for future experiments.

Implementation of this protocol in other laboratories requires determination of two concentrations:

– concentration of bacteriolytic agent that lyse all bacteria in the sample during the experiment, typically with agar plating method (*lysis control)*,
– concentration of fluorescent dye that is not saturated during lysis (*positive control*).

There are three technical requirements to implement the protocol described in this article. If (1) a signal from *lysis control* shows initial increase, (2) reaches a plateau near the end of the assay and (3) signal from *positive control* is around 33% larger than the plateau, the fluorescent protocol can be implemented. Determination of optimal bacteria/bacteriolytic agent concentrations or time/interval of the measurements during the assay improves the reproducibility and precision of the measurements, but still is technically optional. Implementation of the mathematical model is highly beneficial for comparison between other lytic agents and different bacteria concentrations, but still technically optional.

Traditionally, application of Michaelis-Menten calculations for bacteriolytic enzymes has been challenging since the substrate for the endolysin is the peptidoglycan, yet, the measured signals represent either light scattering, or in our case, DNA bound by the fluorescent dye rather than lysed peptidoglycan (Lebaron et al., 1998; Ainsworth, 2014). Michaelis-Menten calculations measure the correlation between concentrations of substrate and the rate of the enzymatic reaction. Moreover, a bacterial cell is not a typical substrate due to the capability to simultaneously bind thousands of endolysin particles despite only a single lytic event “converting” a cell into a signal. Nonetheless, calculations of a Michaelis-Menten model for our data reveals a strong correlation between enzyme activity and a bacteria lysis, thus providing grounds for prediction of enzyme behaviour in comparison to other enzymes, and supporting an earlier hypothesis that a fixed number of bonds in the peptidoglycan need to undergo reaction before the bacteria is lysed (Mitchell et al., 2010). In the opposite case, varying number of bonds required for the lysis would cause high randomness in the model and large errors (Ainsworth, 2014). Of note, our use of mathematical models and calculations is limited to the data available in this study and it requires further testing and validation in other conditions, for instance, the one-phase association model used herein for Pal endolysin. Other attempts to characterise Michaelis-Menten for endolysin characteristics exist, such as the EnzChek^TM^ Lysozyme Assay Kit (Invitrogen) that uses labelled purified peptidoglycan. This commercially available assay, however, does not target the phenomenon of bacteria lysis, instead it detects peptidoglycan-derived substrate hydrolysis by agents of strictly defined specificity (Tafoya et al., 2013).

Sytox Green produces its signal by interaction with released bacterial DNA, but it can also penetrate into metabolically inactive bacteria, so the fluorescent signal is directly correlated to the amount of bacteria that have been inactivated, not necessarily completely disintegrated (Lebaron et al., 1998). Minor damage of the peptidoglycan that renders bacteria inactive but not destroyed cannot be detected by the indirect turbidity reduction assay. Moreover, large particles (if present in the investigated solution) blocking light in the indirect turbidity assay may strongly affect results, but in the fluorometric assay, where the dye itself is the source of light, such particles may have a lesser impact. On the other hand, both fluorometric assay and turbidity reduction assay employ light detection. Thus, they both may have decreased precision in environments that are not fully transparent for specific spectra. Importantly, fluorescent DNA dyes are constantly being developed, including a very wide range of offered spectra (Bucevičius et al., 2018; Okamoto, 2019; Quyen et al., 2019).

This presented approach can be used for rapid detection of susceptible bacteria in a variety of sample matrices or even in a mixture of bacterial strains when a defined selective bacteriolytic agent (e.g., a specific endolysin) is used. Further, with the use of the well-defined specificity of a bacteriolytic agent, bacterial identification can be achieved. Since fluorescent dyes that bind DNA deliver quantitative, real-time measurable signal of bacterial lysis, they have a potential for development of new, precise, high-throughput, low-cost, and rapid tests, applicable even in very low concentration and/or mixtures of bacteria. The World Health Organisation (WHO) and the Centres for Disease Control and Prevention repeatedly called for development of new or alternative antibacterial treatments (World Health Organisation (WHO), 2001; United States Government, 2015; World Health Organisation (WHO), 2015; 2016; 2018a). We propose use of fluorescent dyes and this fluorometric assay as a way to develop and improve methods broadening possibilities in microbiology both in the research of bacteriolytic agents and in the analysis or identification of bacteria samples.

## Data Availability Statement

The original contributions presented in the study are included in the article/Supplementary Material, further inquiries can be directed to the corresponding authors.

## Author Contributions

MH contributed to the conception and design of the study, purification of endolysins, organization and execution of the experiments, statistical analysis, data presentation, and writing the first draft of manuscript. KG contributed to purification of endolysins, organization and immensely helped in execution of the experiments. PM, ZK, and JM contributed to execution of the experiments. BO contributed to purification of endolysins. DN contributed to data presentation, introduced many big corrections to the article and provided others with immense scientific knowledge. KD contributed to organization of the work, data presentation, provided general overview of the works, introduced many correction to the article, added hers immense knowledge, curiosity and patience. All authors contributed to the article and approved the submitted version.

## Conflict of Interest

The authors declare that the research was conducted in the absence of any commercial or financial relationships that could be construed as a potential conflict of interest.

## Publisher’s Note

All claims expressed in this article are solely those of the authors and do not necessarily represent those of their affiliated organizations, or those of the publisher, the editors and the reviewers. Any product that may be evaluated in this article, or claim that may be made by its manufacturer, is not guaranteed or endorsed by the publisher.

## References

[B1] Abdelkader K. Gerstmans H. Saafan A. Dishisha T. Briers Y. (2019). The preclinical and clinical progress of bacteriophages and their lytic enzymes: the parts are easier than the whole. *Viruses* 11:96. 10.3390/v11020096 30678377 PMC6409994

[B2] Ainsworth S. (2014). Practical steady-state enzyme kinetics. *Methods Enzymol.* 536 3–15. 10.1016/b978-0-12-420070-8.00001-5 24423262

[B3] Armstrong G. L. Conn L. A. Pinner R. W. (1999). Trends in infectious disease mortality in the United States during the 20th century. *JAMA* 281 61–66. 10.1001/jama.281.1.61 9892452

[B4] Bucevičius J. Lukinavičius G. Gerasimaite R. (2018). The use of hoechst dyes for DNA staining and beyond. *Chemosensors* 6:18. 10.3390/chemosensors6020018

[B5] Dams D. Briers Y. (2019). “Enzybiotics: enzyme-based antibacterials as therapeutics,” in *Therapeutic Enzymes: Function and Clinical Implications*, ed. Labrou N. (Singapore: Springer Singapore), 233–253. 10.1007/978-981-13-7709-9_11 31482502

[B6] Department of Commerce and Labor B of the C (1906). *Mortalitz Statistics, 1900 to 1904.* Washington, DC: US Department of Commerce and Labor.

[B7] Fischetti V. A. (2001). Phage antibacterials make a comeback. *Nat. Biotechnol.* 19 734–735. 10.1038/90777 11479562

[B8] Fischetti V. A. (2016). *M Protein and Other Surface Proteins on Streptococci Streptococcus pyogenes: Basic Biology to Clinical Manifestations.* Available from: http://www.ncbi.nlm.nih.gov/pubmed/26866233 (accessed May 5, 2021). 26866208

[B9] Fleming A. (1945). *Nobel Lecture: Penicillin.* Available online at: https://www.nobelprize.org/uploads/2018/06/fleming-lecture.pdf (accessed July 1, 2020)

[B10] Harhala M. Nelson D. C. Miernikiewicz P. Heselpoth R. D. Brzezicka B. Majewska J. (2018). Safety studies of pneumococcal endolysins Cpl-1 and Pal. *Viruses* 10:638. 10.3390/v10110638 30445722 PMC6266847

[B11] Heller A. A. Spence D. M. (2019). A rapid method for post-antibiotic bacterial susceptibility testing. *PLoS One* 14:e0210534. 10.1371/journal.pone.0210534 30629681 PMC6328127

[B12] Hernando-Amado S. Coque T. M. Baquero F. Martínez J. L. (2019). Defining and combating antibiotic resistance from One Health and Global Health perspectives. *Nat. Microbiol.* 4 1432–1442. 10.1038/s41564-019-0503-9 31439928

[B13] Hoyert D. L. Kochanek K. D. Murphy S. L. (1999). Deaths: final data for 1997. *Natl. Vital. Stat. Rep.* 47 1–104. 10410536

[B14] Labrou N. (2019). *Therapeutic Enzymes: Function and Clinical Implications.* Berlin: Springer.

[B15] Lebaron P. Catala P. Parthuisot N. (1998). Effectiveness of SYTOX green stain for bacterial viability assessment. *Appl. Environ. Microbiol.* 64 2697–2700. 10.1128/aem.64.7.2697-2700.1998 9647851 PMC106447

[B16] Lewis C. L. Craig C. C. Senecal A. G. (2014). Mass and density measurements of live and dead gram-negative and gram-positive bacterial populations. *Appl. Environ. Microbiol.* 80 3622–3631. 10.1128/aem.00117-14 24705320 PMC4054131

[B17] Liu Y. Li R. Xiao X. Wang Z. (2019). Molecules that inhibit bacterial resistance enzymes. *Molecules* 24:43. 10.3390/molecules24010043 30583527 PMC6337270

[B18] Loeffler J. M. Djurkovic S. Fischetti V. A. (2003). Phage lytic enzyme Cpl-1 as a novel antimicrobial for pneumococcal bacteremia. *Infect. Immun.* 71 6199–6204. 10.1128/iai.71.11.6199-6204.2003 14573637 PMC219578

[B19] McKellar R. C. Knight K. (2000). A combined discrete-continuous model describing the lag phase of Listeria monocytogenes. *Int. J. Food. Microbiol.* 54 171–180. 10.1016/s0168-1605(99)00204-4 10777067

[B20] Meyers A. Furtmann C. Jose J. (2018). Direct optical density determination of bacterial cultures in microplates for high-throughput screening applications. *Enzyme Microb. Technol.* 118 1–5. 10.1016/j.enzmictec.2018.06.016 30143192

[B21] Mitchell G. J. Nelson D. C. Weitz J. S. (2010). Quantifying enzymatic lysis: estimating the combined effects of chemistry, physiology and physics. *Phys. Biol.* 7:046002. 10.1088/1478-3975/7/4/046002 20921589

[B22] Nikaido H. (2009). Multidrug resistance in bacteria. *Annu. Rev. Biochem.* 78 119–146. 19231985 10.1146/annurev.biochem.78.082907.145923 PMC2839888

[B23] Okamoto A. (2019). Next-generation fluorescent nucleic acids probes for microscopic analysis of intracellular nucleic acids. *Appl. Microsci.* 49 1–7. 10.1186/s42649-019-0017-1 PMC7818349 33580316

[B24] Quyen T. L. Ngo T. A. Bang D. D. Madsen M. Wolff A. (2019). Classification of multiple DNA dyes based on inhibition effects on real-time loop-mediated isothermal amplification (LAMP): prospect for point of care setting. *Front. Microbiol.* 10:1–12. 31681184 10.3389/fmicb.2019.02234 PMC6803449

[B25] Schmelcher M. Loessner M. J. (2021). Bacteriophage endolysinsextending their application to tissues and the bloodstream. *Curr. Opin. Biotechnol.* 68 51–59. 10.1016/j.copbio.2020.09.012 33126104

[B26] Song M. Liu Y. Huang X. Ding S. Wang Y. Shen J. (2020). A broad-spectrum antibiotic adjuvant reverses multidrug-resistant Gram-negative pathogens. *Nat. Microbiol.* 5 1040–1050. 10.1038/s41564-020-0723-z 32424338

[B27] Tafoya D. A. Hildenbrand Z. L. Herrera N. Molugu S. K. Mesyanzhinov V. V. Miroshnikov K. A. (2013). Enzymatic characterization of a lysin encoded by bacteriophage EL. *Bacteriophage* 3:e25449. 10.4161/bact.25449 24228221 PMC3821690

[B28] United States Government (2015). *National Action Plan for Combating Antibiotic-resistant Bacteria.* Washington, DC: United States Government.

[B29] World Health Organisation (WHO) (2001). *WHO Global Strategy for Containment of Antimicrobial Resistance, World Health Organisatin.* Switzerland: World Health Organisation.

[B30] World Health Organisation (WHO) (2015). *Global Action Plan on Antimicrobial Resistance*, Vol. 10. Geneva: World Health Organisation.

[B31] World Health Organisation (WHO) (2018b). *Global Health Estimates 2016 Disease burden bz Cause, Age, Sex, bz Countrz and bz Region, 2000+2016.* Available online at: https://www.who.int/healthinfo/global_burden_disease/estimates/en/index1.html (accessed April 8, 2021).

[B32] World Health Organisation (WHO) (2018a). *Global Framework for Development & Stewardship to Combat Antimicrobial Resistance.* Available online at: https://www.who.int/phi/news/WHO_OIE_FAO_UNEP_Working_paper_of_the_framework_FINAL.pdf (accessed April 8, 2021).

[B33] World Health Organisation [WHO] (2019). *Antibacterial Agents in Clinical Development.* Geneva: World Health Organisation (WHO).

[B34] World Health Organisation (WHO), Food and Agriculture Organization of the United Nations (FAO), and World Organisation for Animal Health (OIE) (2016). *Antimicrobial Resistance**: A Manual for Developing National Action Plans.* Geneva: JAMA.

[B35] Young R. (1992). Bacteriophage lysis: mechanism and regulation. *Microbiol. Mol. Biol. Rev.* 56 430–481. 10.1128/mr.56.3.430-481.1992 1406491 PMC372879

[B36] Yu A. C. S. Loo J. F. C. Yu S. Kong S. K. Chan T. F. (2014). Monitoring bacterial growth using tunable resistive pulse sensing with a pore-based technique. *Appl. Microbiol. Biotechnol.* 98 855–862. 10.1007/s00253-013-5377-9 24287933

