# Supplementary material for: DNA Dye Sytox Green in Detection of Bacteriolytic Activity: High Speed, Precision and Sensitivity Demonstrated With Endolysins
Source: Front Microbiol. 2021 Oct 25;12:752282. doi: 10.3389/fmicb.2021.752282 (PMC8575126; doi:10.3389/fmicb.2021.752282)
Supplement: Supplementary file 1 [file Data_Sheet_1.PDF]

# *Supplementary Material*

## 1 Supplementary Data

The "lytic activity" refers to the initial velocity of lysis in terms of enzyme kinetics and is calculated by the selected mathematical models (see below). If a lytic component with known activity is mixed with an unknown amount of bacteria, the lytic activity value can be called a susceptibility to lysis. This setup can be used for characterisation of bacteria mixtures and analysis of unknown samples, such as clinical or environmental isolates. Representative raw and normalized data for fluorometric and turbidity reduction assays are presented in **Supplementary Figure S1**.

In this study, the Cpl-1 endolysin demonstrated a constant *lytic activity* that fulfils requirements of a linear regression model; this was observed for both the fluorometric and turbidity reduction assays. Of note, the linear characteristics do not apply to the whole lytic curve. Herein, the linear regression model was calculated from progress values 0.2 to 0.75, and it displayed high linearity in both turbidity reduction and fluorometric assay ($R^2$>0.99 for both models) (**Supplementary Figure S2 and Table S2**). This means that the *lytic activity* was constant between 0.2 and 75, but the reaction slowed down when more than 75% fraction of bacteria was lysed. This was most likely due to substrate depletion. Points below *progress* equal to 0.2 were not applicable for linear regression model probably due to a delay in a reaction start. *Lytic activity* in this model can be evaluated by the slope coefficient. Graphical representation of both methods with the use of the mathematical model is shown in **Supplementary Figure S2 and Table S2**.

In contrast to Cpl-1, the Pal endolysin data did not fit a linear regression model for either method. For concentrations of the Pal endolysin from 1 mg/L to 110 mg/L, a one-phase association model best fit the data (GraphPad Prism 7; $R^2$>0,99 for both measurement methods). Representative curves are shown in **Supplementary Figure S1** and **Supplementary Figure S2**. A one-phase association mathematically describes a type of reaction where the change in the lysed fraction of bacteria is highest at the beginning and gradually drops to zero when all bacteria are lysed. *Lytic activity* in this model can be evaluated by a K value, which depicts change of *progress* in time at the beginning of the reaction (initial velocity). In low concentrations of the bacteriolytic agent (below 1 mg/L of Pal and below 2 mg/L for Cpl-1) all measured points were used for calculation of linear regression due to insufficient lysis in 10 min assay. Comparison of mathematical models are summarised in **Supplementary Table S2**.

**Sytox Green demonstrates the best applicability for detection of bacterial lysis when compared to DAPI, SYTO 9 and propidium iodide**

We selected the DNA dye suitable for the fluorometric assay with following assumptions:

1) A suitable DNA dye should provide a stable, linear signal for the negative control (bacterial sample, see Materials and Methods for details) as determined by a linear regression model. If the signal from the negative control (bacteria, without endolysin) changes over time, a linear regression model evaluates a coefficient of determination ($R^2$ value) and a statistical significance of the model showing probability that this measured change in time (slope) is accidental. A coefficient of determination ($R^2$), specifies the goodness-of-fit, that is it describes how much the developed model varies from the experimental data (for the perfect fit $R^2$ equal to 1.0). The statistical significance of the model is expressed as a p-value, where $p<0.05$ means that the slope deviates statistically significantly from zero.
2) The DNA dye should enable a detection of a fluorescent signal reaching a plateau after the lysis reaction ends during the reaction time. This plateau was used later as the *max* signal (progress equal to 1.0, meaning lysis of all the cells in the sample). The same plateau is seen in the turbidity reduction assay. The DNA dye showing practically no change in the last 3 min of the measurement was deemed suitable for our purposes.
3) A suitable DNA dye should show high responsiveness, meaning it rapidly enters dead cells binding the bacterial DNA, preferentially not requiring complete lysis of the cells by the bacteriolytic agent. The DNA dye showing the most rapid change of the fluorescent signal in response to a bacteriolytic agent added to a sample may provide the highest speed and precision of measurements. We evaluated this by comparison of minimal time required to detect the change of fluorescent signal equal to 50% of the total detected change.

We tested four fluorescent DNA dyes offered in two commercially available kits, Sytox Green and DAPI from the ViaGram™ Red+ bacterial Gram Stain and Viability Kit (ThermoFisher Scientific), and SYTO9 and propidium iodide from the LIVE/DEAD® BacLight™ Bacterial Viability Kit (ThermoFisher Scientific). DAPI and SYTO9 DNA dyes are used commonly to stain live cells. Sytox Green and propidium iodide are used to stain metabolically inactive cells, so a rise of the signal after addition of the endolysin is expected due to the increase in metabolically inactive cells.

Comparison of propidium iodide, SYTO9, Sytox Green and DAPI proved that Sytox Green was the most suitable for our purpose of bacterial lysis detection. For the other dyes, the coefficient of determination was higher ($R^2$=0.047 for Sytox Green, $R^2$>0.3 for other dyes) and the statistical significance of the slope being accidental is highest (slope = 0; p=0.21 for Sytox Green, p<0.001 for other dyes). The plateau (*max* signal) was detected only for DAPI and Sytox Green. DAPI required almost 6 minutes to reach 50% of the measured reaction progress and Sytox Green required only 4 minutes, making this dye as the best choice for further experimentation. A detailed description in Supplementary Table S3.

## 2 Supplementary Figures and Tables

## 2.1 Supplementary Figures

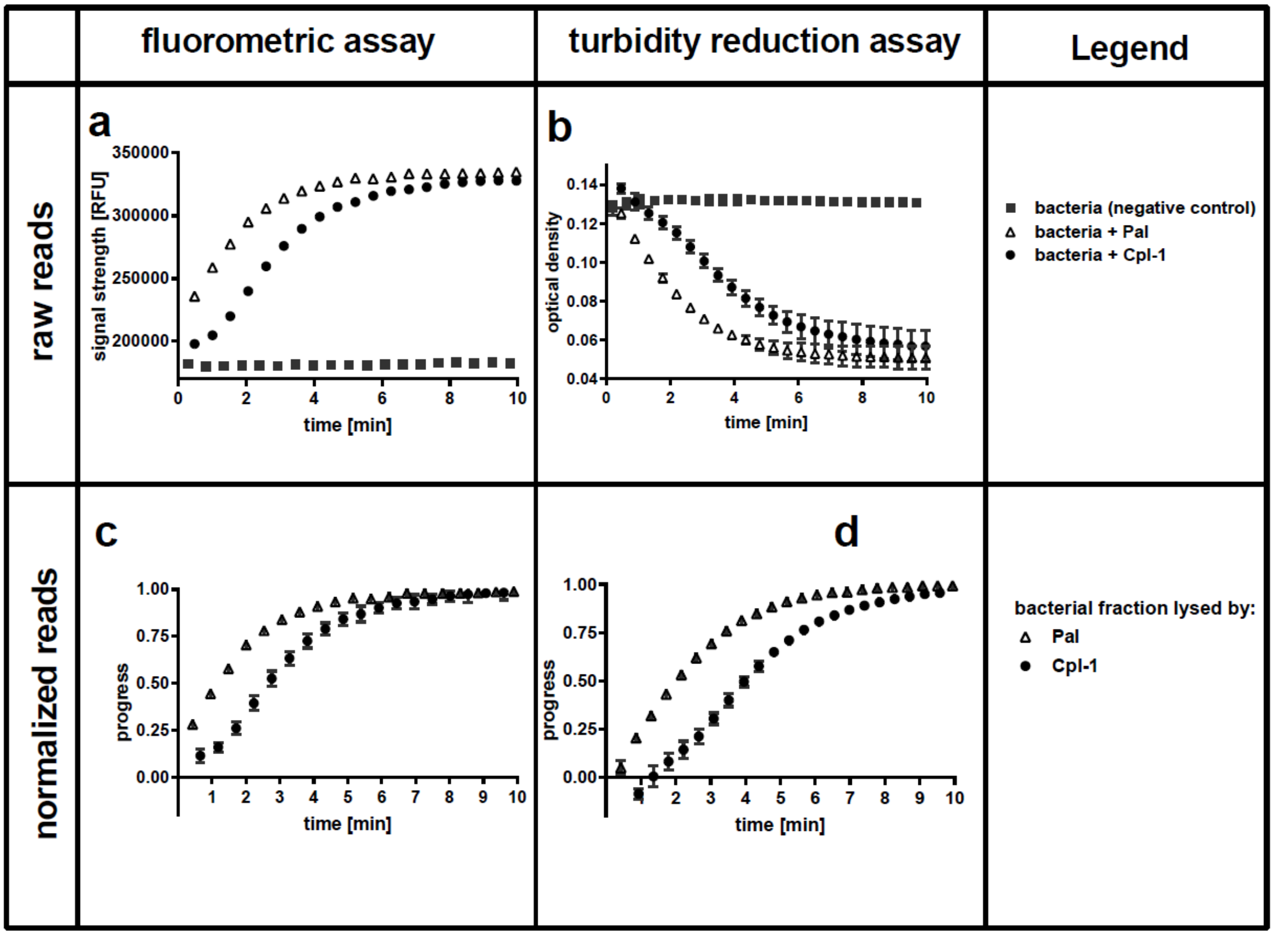


**Supplementary Figure S1. Fluorometric and turbidity reduction assays in bacterial lysis detection: representative reads and normalized reads.** Bacterial lysis was detected in PBS (200 µl) with $1.6 \times 10^7$ CFU, Cpl-1 or Pal (final concentrations of 46 mg/L and 13 mg/L, respectively) and 0.25% DMSO or Sytox Green solution (turbidity reduction or fluorometric assay respectively, see Materials and Methods for details). Reads measured in fluorometric (**a**) and turbidity assays (**b**). Data normalization: the bacterial fraction lysed (progress) calculated from reads for fluorometric (**c**) and turbidity reduction assays (**d**) (see: Equation 1; progress equal 0 means no lysis and progress equal 1 means all bacteria are lysed, for details see Materials and Methods). Average values from three technical repeats are demonstrated, whiskers represent the standard deviation.

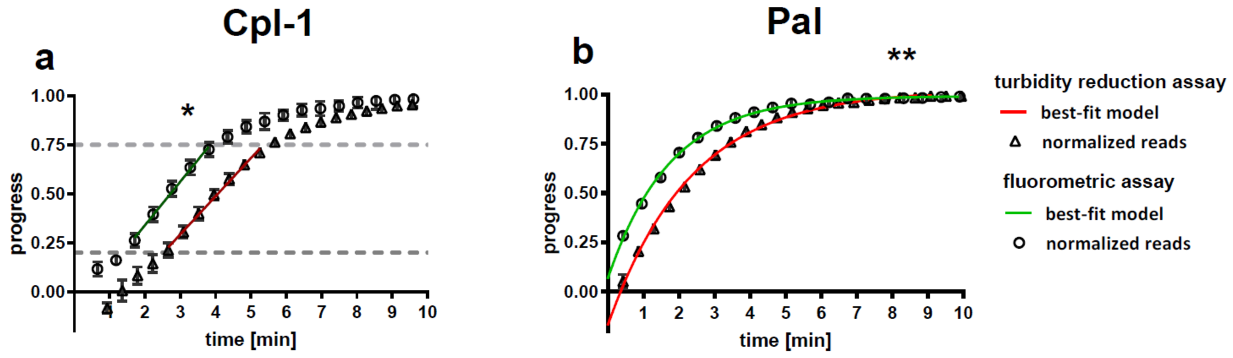


**Supplementary Figure S2. Fitted mathematical models for Cpl-1 and Pal lytic curves.** Bacterial lysis was detected in PBS (200 µL) with $1.6 \times 10^7$ CFU, Cpl-1 or Pal (final concentrations of 46 mg/L and 13 mg/L, respectively) and 0.25% DMSO or Sytox Green solution (turbidity reduction or fluorometric assay respectively). Graphical representation of mathematical models calculated from data presented in Supplementary Figure S1 for Cpl-1 (**a**) and Pal (**b**). Proposed best-fit models allow for highly accurate ($R^2$>0.99 in each model) and statistically significant (probability of inadequate model, p<0.0002, GraphPad Prism 7). Average values from three technical repeats are demonstrated and whiskers represent the standard deviation. Points between grey lines were used for the linear regression model of the Cpl-1 endolysin. All points measured for the Pal endolysin were used for mathematical modelling (see Materials and Methods for details). * the significant difference between lytic activity detected in fluorometric and turbidity reduction assays for Cpl-1 (p=0.048); ** the significant difference between lytic activity detected in fluorometric and turbidity reduction assays for Pal (p<0.0001).

**b**

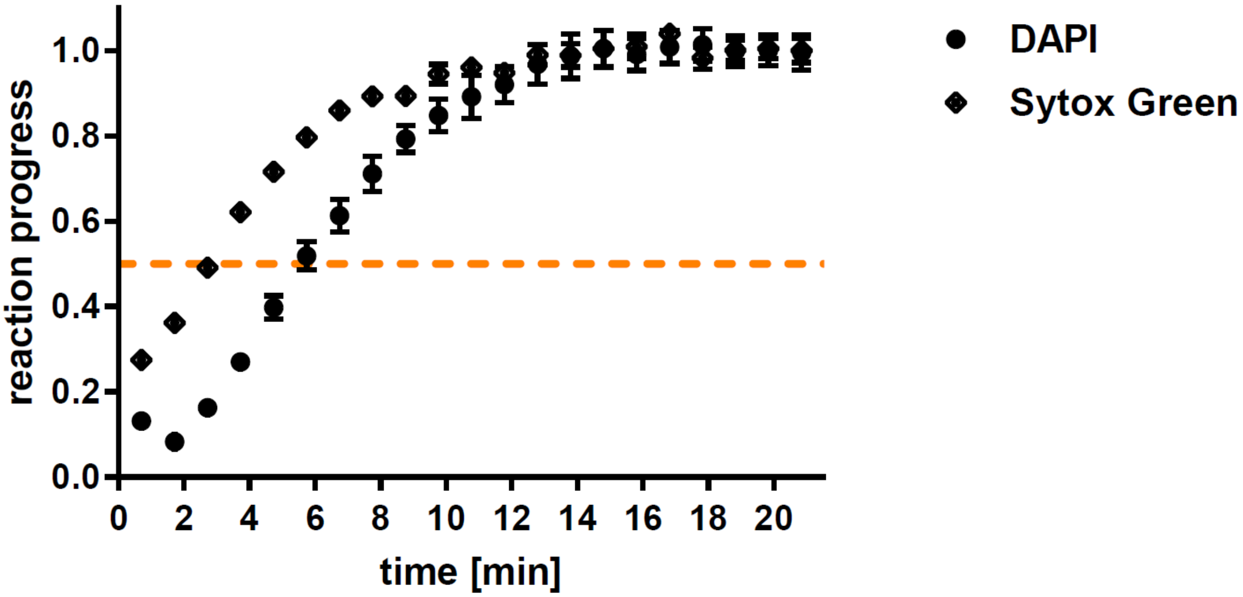


**Supplementary Figure S3. Comparison of DAPI and Sytox Green in detection of bacterial lysis.** *Progress* of bacterial lysis (fraction of lysed bacteria) by the Cpl-1 endolysin (50 mg/L) measured with two dyes (DAPI and Sytox Green). 0.0 is the fluorescence signal of bacteria-only control; 1.0 is complete lysis.

## 2.2 Supplementary Tables

| Pal conc. [mg/L] | Turbidity reduction assay | | | Fluorometric assay | | |
|---|---|---|---|---|---|---|
| | Adjusted R square | K | Std. Error of K | Adjusted R square | K | Std. Error of K |
| 110 | 0.936 | 0.858 | 0.041 | 0.885 | 1.259 | 0.103 |
| 55 | 0.976 | 0.765 | 0.022 | 0.977 | 0.987 | 0.033 |
| 27.5 | 0.982 | 0.635 | 0.017 | 0.991 | 0.762 | 0.017 |
| 13.7 | 0.982 | 0.492 | 0.014 | 0.957 | 0.603 | 0.030 |
| 6.9 | 0.945 | 0.328 | 0.021 | 0.994 | 0.447 | 0.010 |
| 3.44 | 0.982 | 0.157 | 0.010 | 0.985 | 0.280 | 0.012 |
| 1.72 | 0.970 | 0.052 | 0.013 | 0.921 | 0.166 | 0.027 |

**Supplementary Table S1. Major values calculated for one-phase association model of Pal activity.** Comparison of an adjusted R value, K value, and Standard error of K value between both measurement methods calculated during evaluation of the one phase association model as best-fit model for Pal endolysin.

| Pal | | | Cpl-1 | | |
|---|---|---|---|---|---|
| **Comparison of Fits** | | | | | |
| **Best-fit values** | **Turbidity reduction assay** | **Fluorometric assay** | **Best-fit values** | **Turbidity reduction assay** | **Fluorometric assay** |
| Y0 | -0.2997 | -0.06351 | Slope | 0.1947 | 0.2222 |
| Plateau | 1.017 | 0.9932 | Y-intercept | -0.2915 | -0.1048 |
| K | 0.4898 | 0.5754 | X-intercept | 1.497 | 0.4719 |
| **95% CI (profile likelihood)** | | | | | |
| Y0 | -0.3378 to -0.2626 | -0.1002 to -0.02811 | Slope | 0.1775 to 0.212 | 0.1904 to 0.2539 |
| Plateau | 1.006 to 1.027 | 0.9855 to 1.001 | X-intercept | 1.245 to 1.71 | 0.07261 to 0.7754 |
| K | 0.4677 to 0.5124 | 0.5475 to 0.6041 | **Y-intercept** | -0.3613 to -0.2216 | -0.1957 to -0.01391 |
| **Goodness of Fit** | | | | | |
| $R^2$ | 0.9984 | 0.9985 | $R^2$ | 0.9941 | 0.994 |
| **Are slopes equal?** | | | | | |
| **P value** | **<0.0001** | | **0.048** | | |
| Significant? | **Differences between slopes are significant** | | **Differences between slopes are significant** | | |

**Supplementary Table S2. Statistical analysis of mathematical models used to evaluate activity of enzymes for Supplementary Figure S2.** Comparison of best-fit models based on representative data in **Supplementary Figure S1** and **Supplementary Figure S2**. A linear regression model is the best fit model for the Cpl-1 endolysin and one-phase association is the best fit model for the Pal

endolysin. For the Cpl-1 endolysin, a linear fragment for progress between 0.2 and 0.75 is suitable for calculation of lytic activity ($R^2 > 0.99$ for both methods). Large differences in values of the X and Y-intercept indicate significant delay in the detected signal for the turbidity reduction method. For the Pal endolysin, a one-phase association model ($R^2 > 0.99$ for both methods, calculated by GraphPad Prism 7) is the best-fit due to a lack of detected slope. There is a highly significant difference between the calculated K values between assays ($p<0.0001$). Similar to Cpl-1, there are also significant differences in the Y - intercept values for Pal, presumably due to the delay detected in the turbidity reduction assay.

| | | SYTO9 | DAPI | PI | Sytox Green |
|---|---|---|---|---|---|
| linearity of the negative control | slope | 304 | 195 | 14.1 | -5.61 |
| | $R^2$ | 0.35 | 0.98 | 0.53 | 0.046 |
| | p; NH: slope=0 | 0.0006 | <0.0001 | <0.0001 | 0.21 |
| | Is deviation from 0 significant? | Yes | Yes | Yes | No |
| after-reaction plateau | slope | -624 | 149 | 288 | -40.8 |
| | R2 | 0.99 | 0.70 | 0.99 | 0.044 |
| | p; NH: slope=0 | 0.0007 | 0.074 | 0.0002 | 0.73 |
| | Is deviation from 0 significant? | Yes | No | Yes | No |
| responsiveness | time to reach 50% progress | Not available | <6 min | Not available | <4min |

**Supplementary Table S3**: The table shows a statistical evaluation of four different DNA dyes: SYTO9, DAPI, propidium iodide (PI) and Sytox Green. The four dyes were tested for stability in a bacteria-only control, reaching the plateau (>15 min) and responsiveness. The plateau associated with destruction of the vast majority of bacteria was evaluated with linear regression model. Sytox Green was the only one showing stability and linearity of the negative control, ability to detect a plateau after reaction and a high responsiveness. By process of elimination, Sytox Green was used for the remainder of the experiments. For the above studies, Cpl-1 was used in a concentration of 50 mg/L. The coefficient of determination (R2) shows linearity of the slope, where low values demonstrate stability of the measured raw signal, which is required in both the negative control and after reaction plateau, whereas high values show gradual, stable change of the raw signal over time.; p – p-values between slope equal to 0 and a calculated one; NH - null hypothesis.

| **Bacteria** | **lytic activity [min$^{-1}$]** | **SD** |
|---|---|---|
| 1.64E+07 | 0.18 | 0.019 |
| 1.15E+07 | 0.19 | 0.031 |
| 8.20E+06 | 0.19 | 0.030 |
| 4.92E+06 | 0.20 | 0.030 |
| 1.64E+06 | 0.20 | 0.008 |
| 4.92E+05 | 0.20 | 0.025 |
| 1.64E+05 | 0.21 | 0.020 |
| 5.41E+04 | 0.21 | 0.021 |
| 1.80E+04 | 0.18 | 0.025 |
| 6.00E+03 | 0.16 | 0.075 |
| 1.97E+03 | 0.17 | 0.042 |
| 6.72E+02 | 0.11 | 0.061 |

**Supplementary Table S4.** Lytic activity measured for varying concentrations of bacteria for experiments presented in Fig. 3 a and b.

| Line | | One-phase association | |
|---|---|---|---|
| **Best-fit values** | | | |
| Y0 | 0.1363 | Y0 | -0.08919 |
| Slope | 0.1397 | Plateau | 1.104 |
| | | K | 0.2844 |
| | | Tau | 3.516 |
| | | Half-time | 2.437 |
| | | Span | 1.193 |
| **Std. Error** | | | |
| Y0 | 0.05232 | Y0 | 0.07494 |
| Slope | 0.01782 | Plateau | 0.05705 |
| | | K | 0.04653 |
| | | Span | 0.05858 |
| **Goodness of Fit** | | | |
| Degrees of Freedom | 38 | Degrees of Freedom | 102 |
| R square | 0.6181 | R square | 0.8065 |
| Adjusted R square | 0.608 | Adjusted R square | 0.8027 |

**Supplementary Table S5. Comparison of mathematical models used to evaluate the activity of the Cpl-1 phage endolysin in the lowest concentration of bacteria.** Results of our statistical analysis of best-fit models for a bacterial concentration of 680 CFU/sample. The linear fragment is calculated for data with progress between 0.2 and 0.75. The one-phase association model was calculated from all points within the 10 min analysis.
